# Whole-body bone mineral density and markers of bone homeostasis in adults with normal-weight obesity

**DOI:** 10.1016/j.obpill.2023.100073

**Published:** 2023-06-01

**Authors:** Bryant H. Keirns, Christina M. Sciarrillo, Austin R. Medlin, Samantha M. Hart, Elyse M. Cronic, Sam R. Emerson

**Affiliations:** a Department of Nutritional Sciences, Oklahoma State University 301 Nancy Randolph Davis, Stillwater, OK, 74078, USA; b Department of Health & Wellness Design, Indiana University School of Public Health, 1025 E. Seventh St., Bloomington, IN, 47405, USA

**Keywords:** Normal-weight obesity, Bone mineral density, Bone formation, Bone resorption, Body fat percent

## Abstract

**Background:**

Normal-weight obesity (NWO) describes individuals with a normal body mass index (BMI), but high body fat percent. NWO are at-risk for cardiometabolic diseases, but little is known about their bone health.

**Methods:**

Adults (N = 24) were classified as NWO (n = 12; 5M/7F) or low body fat percent controls (Con; n = 12; 6M/6F). Body composition and whole-body bone mineral density (BMD) were assessed using DXA. A serum bioplex assay was performed to examine markers related to bone formation and resorption.

**Results:**

In addition to higher body fat percent and visceral fat, NWO had lower whole-body BMD relative to Con (*p*'s < 0.05). Circulating leptin was higher in NWO than Con (*p* < 0.05). Two biomarkers generally associated with lower bone mass – sclerostin and parathyroid hormone – were higher in NWO compared to Con (*p*'s < 0.05).

**Conclusion:**

In this preliminary study, adults with NWO displayed lower whole-body BMD alongside evidence of bone resorption. Impaired bone health may be another subclinical risk factor present in NWO.

## 1 Introduction

Low bone mass leading to osteoporosis remains a significant health issue, with ∼9 million bone fractures attributable to osteoporosis occurring worldwide each year [1]. Although a complex relationship, there is evidence that obesity is one factor that negatively impacts bone through several mechanisms (e.g., increased direction of mesenchymal stem cells toward adipocytes over osteoblasts, increased low-grade inflammation) [2]. To date, most research examining the relationship between bone and increased adiposity has focused on obesity in the classical sense (e.g., body mass index or BMI >30 kg/m^2^). However, emerging research has suggested that “normal-weight obesity” (NWO) – a state characterized by a normal BMI, but high body fat percent – may also be linked to poor bone health [3]. Specifically, children/adolescents with NWO display poor skeletal robustness versus lower body fat peers [4] and body fat percent was negatively associated with femoral bone mineral density (BMD) in an adult cohort of normal BMI individuals that was 71% NWO [5]. Despite evidence that bone may be altered in NWO, serum indicators that could provide insights on what aspect(s) of bone metabolism is affected (i.e., accrual and/or resorption) remain unexamined. Consistent with this growing literature, we observed lower whole-body BMD in adults with NWO compared to lower body fat controls (Con) in recently completed studies. Therefore, we followed up on this BMD difference by measuring serum markers associated with bone metabolism to preliminarily investigate potential mechanisms related to the reported poor bone health in NWO.

## 2 Methods

### 2.1 Participants

Participants (N = 24; aged 19-45y) in the present report had recently completed one of two studies in our laboratory. The first study (n = 20/24 participants) examined cardiovascular disease risk factors in NWO (i.e., postprandial triglycerides, flow-mediated dilation, serum cytokines; NCT05008952). The second study (n = 4/24 participants) evaluated the validity of an abbreviated fat tolerance test in more general populations (*unpublished*). Recruitment for these studies took place using mass email, social media, flyers, and word of mouth. Interested individuals with a BMI in the normal range (i.e., 18.5-24.9 kg/m^2^) were invited to the laboratory to estimate body fat percentage using bioimpedance (SECA; Hamburg, Germany) and then grouped as either NWO or Con. Body fat percentage was later confirmed using DXA. NWO (n = 12; 5M/7F) was defined as a BMI of 18.5-24.9 kg/m^2^ and body fat percent ≥25% (M) or ≥ 35% (F). Similar body fat percent cutoffs have previously been used to define NWO and the World Health Organization has endorsed these cutoffs as consistent with obesity [[6], [7], [8]]. Con was defined as a BMI of 18.5-24.9 kg/m^2^ and body fat percent <25% (M) or < 35% (F). Participants did not have known cardiometabolic or inflammatory conditions, were premenopausal, and did not use tobacco products, lipid-lowering drugs, or anti-inflammatory medications. This study was conducted according to the guidelines laid down in the Declaration of Helsinki and all procedures involving human participants were approved by the Oklahoma State University Institutional Review Board (IRB-20-339-STW). Informed consent was obtained from all participants.

### 2.2 Body composition and bone density assessment

Body fat and lean mass parameters and whole-body BMD were assessed with DXA (Hologic; Marlborough, MA).

### 2.3 Serum analyses

Fasting venous blood samples were obtained from each participant in the morning between 0700 and 1000. A metabolic panel was performed immediately using the Piccolo Xpress clinical chemistry analyzer (Abbott; Chicago, IL). Other blood samples were allowed to clot for 30 min, centrifuged at 2500×*g* ​for 15 min at 4 °C to obtain serum, and then stored at −80 °C. Serum markers related to skeletal health were assessed with a bone discovery bioplex (Millipore; Burlington, MA) conducted by Eve Technologies (Calgary, AB, Canada) according to manufacturer instructions. The panel included markers generally related to bone formation and maintenance such as adrenocorticotropic hormone, osteoprotegerin, osteocalcin, and leptin, as well as markers considered to reflect low bone quality and/or resorption including dickkopf-related protein (DKK)-1, sclerostin, fibroblast growth factor (FGF)-23, osteopontin, parathyroid hormone, and tumor necrosis factor (TNF)-α.

### 2.4 Statistical analyses

The present secondary analysis tested the hypothesis that serum indicators of bone health would be altered in NWO versus lower body fat controls. Data were analyzed in GraphPad Prism 8.0 (GraphPad Prism Inc.; La Jolla, CA). The ROUT test was used to identify extreme outliers [9]. After testing for normality with the Shapiro-Wilks test, group differences were assessed using Student's t-tests. All data are presented as mean ± SD (α = 0.05).

## 3 Results

General participant characteristics, blood pressure, and metabolic markers are presented in Table 1. Our preliminary observation that NWO adults displayed lower whole-body BMD versus Con is presented in Table 2 (*p* < 0.05). DXA results also revealed that NWO displayed higher body fat percent, absolute fat mass, and visceral fat as well as lower lean mass percent compared to Con (Table 2; *p*'s < 0.01).

**Table 1:** General participant characteristics.

	Con	NWO
Age (years)	28 ± 5	32 ± 9
Sex		
*Male*	6/12	5/12
*Female*	6/12	7/12
Ethnicity		
*Caucasian*	11/12	8/12
*Asian*	0/12	3/12
*Hispanic*	1/12	1/12
Mass (kg)	69.3 ± 11.1	70.8 ± 9.3
BMI (kg/m^2^)	22.4 ± 1.7	24.2 ± 1.0
WC (inches)	29.6 ± 2.8	32.9 ± 3.4
Systolic BP (mmHg)	109 ± 11	107 ± 9
Diastolic BP (mmHg)	74 ± 8	76 ± 7
Glucose (mg/dL)	93.7 ± 6.8	96.4 ± 2.9
Total-C (mg/dL)	157.8 ± 31.6	170.1 ± 45.3
HDL-C (mg/dL)	65.3 ± 10.7	58.0 ± 10.1
LDL-C (mg/dL)	78.5 ± 27.3	93.7 ± 38.2
VLDL-C (mg/dL)	13.8 ± 4.8	18.3 ± 4.6
Non-HDL-C (mg/dL)	92.4 ± 28.5	112.1 ± 40.8
Triglycerides (mg/dL)	69.5 ± 24.4	91.0 ± 23.0
ALT (U/L)	22.7 ± 5.6	25.1 ± 6.7
AST (U/L)	26.4 ± 5.7	23.3 ± 3.7

**Table 2:** Body composition and whole-body bone mineral density assessment.

	Con	NWO	*p*-value
Body Fat (%)	24.2 ± 5.8	33.4 ​± ​4.5∗	**0.0003**
Body Fat (kg)	16.7 ± 4.1	23.5 ​± ​2.6∗	**<0.0001**
VAT (g)	250.2 ± 81.7	395.9 ​± ​131.5∗	**0.0036**
Lean mass (%)	73.2 ± 6.2	63.4 ​± ​4.4∗	**0.0002**
Lean mass (kg)	50.9 ± 10.4	45.1 ± 8.3	0.1782
Whole-body BMD (g/cm^2^)	1.195 ± 0.118	1.104 ​± ​0.080∗	**0.0359**

All metabolic parameters presented are fasting. Data are presented as mean ± SD. Abbreviations: **Con** healthy control; **NWO** normal-weight obesity; **BMI** body mass index; **WC** waist circumference; **BP** blood pressure; **HDL-C** high density lipoprotein cholesterol; **LDL-C** low density lipoprotein cholesterol; **VLDL-C** very low density lipoprotein cholesterol; **TC** total cholesterol; **ALT** alanine transaminase; **AST** aspartate aminotransferase.

Data are presented as mean ​± ​SD. ∗ denotes a statistically significant difference between groups. Abbreviations: **Con** healthy control; **NWO** normal-weight obesity; **VAT** visceral adipose tissue; **BMD** bone mineral density.

**Fig. 1:**
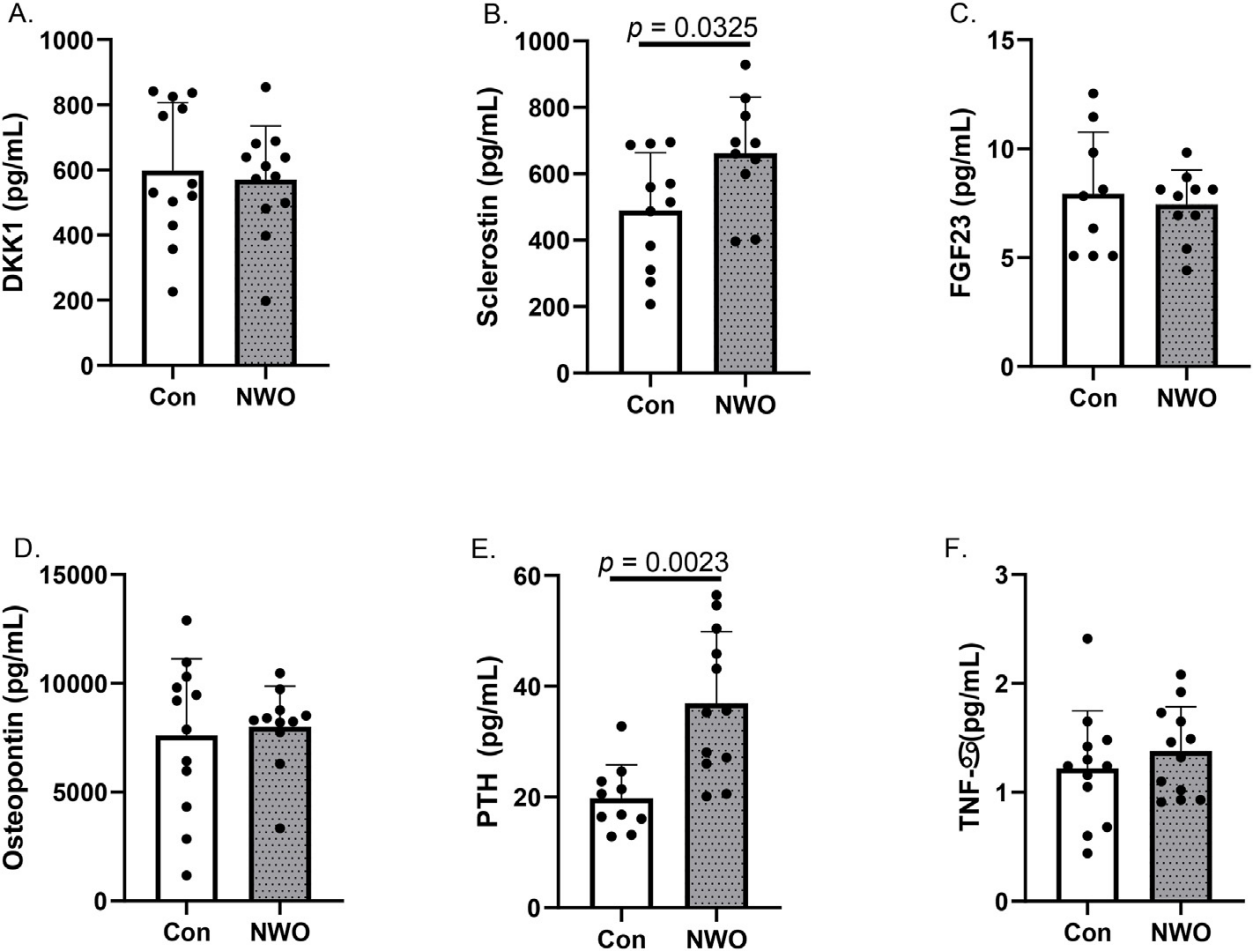
**Biomarkers Associated with Bone Resorption. (A)** DDK1, **(B)** sclerostin, **(C)** FGF23, **(D)** osteopontin, **(E)** PTH, and **(F)** TNF-α. Data are presented as mean ± SD. *p*-values are displayed when statistically significant differences were observed between NWO and Con. Abbreviations: **Con** control; **NWO** normal-weight obesity; **DKK** dickkopf-related protein**; FGF** fibroblast growth factor; **PTH** parathyroid hormone; **TNF** tumor necrosis factor.

**Fig. 2:**
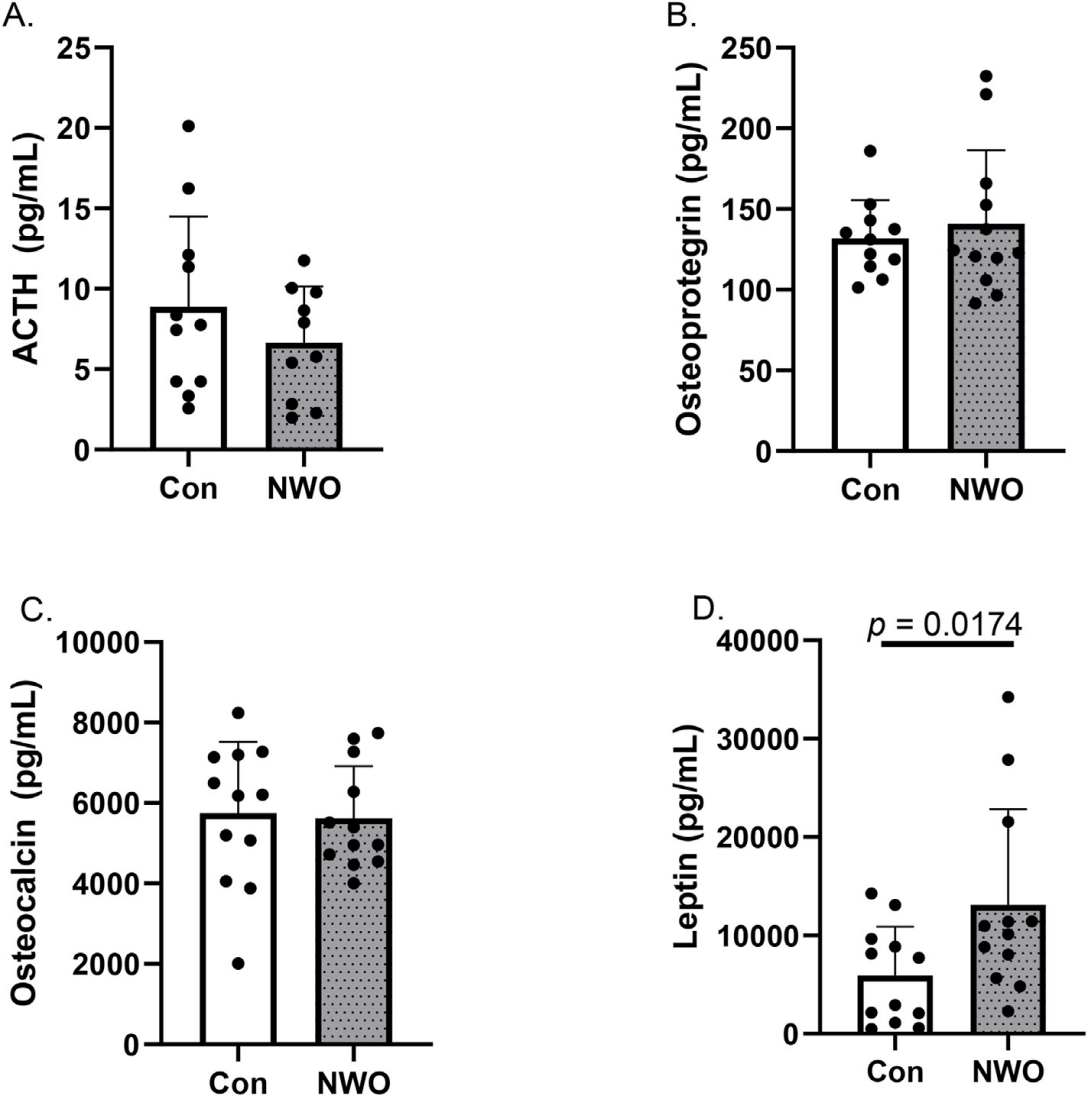
**Biomarkers Associated with Bone Formation. (A)** ACTH, **(B)** osteoprotegrin, **(C)** osteocalcin, **(D)** leptin. Data are presented as mean ± SD. *p*-values are displayed when statistically significant differences were observed between NWO and Con. Abbreviations: **Con** control; **NWO** normal-weight obesity; **ACTH** adrenocorticotropic hormone.

When assessing biomarkers often associated with lower bone mass, we observed that sclerostin and parathyroid hormone were greater in NWO relative to Con (Fig. 1, Fig. 2; *p*'s < 0.05). All other markers related to lower bone mass were not different between groups. Leptin, which is commonly implicated in bone maintenance, was higher in NWO compared to Con (Fig. 2D; *p* < 0.05). Other serum markers reflecting bone formation or maintenance were not different between Con and NWO (Fig. 2A-C).

## 4 Discussion

The present study was the first to our knowledge to examine circulating biomarkers associated with bone metabolism in NWO. This study was motivated by the observation that our NWO sample displayed subclinically lower whole-body BMD than counterparts with low body fat percent. In addition to low BMD, we observed higher parathyroid hormone and sclerostin – two negative regulators of bone mass – in NWO. These data are consistent with data in children linking NWO to poor lower extremity skeletal robustness (i.e., low ratio of femur epicondyle breadth to height) [4]. Two other reports in adolescents [10] and collegiate dancers [11] with NWO reported that whole body BMD is similar to controls, but our NWO group was older (mean age of 32 years) and it is conceivable that it may take more time for a lower BMD phenotype to develop in adults with NWO (and thus not be detected in these younger populations). However, larger studies are needed to test this hypothesis and confirm our findings.

With respect to markers associated with bone formation/maintenance, we observed increased leptin in NWO relative to Con. From an adipose tissue physiology standpoint, this finding is unsurprising, as our NWO group had 41% greater fat mass than Con and leptin correlates with total fat mass [12]. When taking a bone-centric view of these data, higher circulating leptin would seem in contrast to lower BMD in NWO given leptin's role in bone maintenance. However, the impact of obesity on bone health appears to have both positive (e.g., increased leptin, mechanical loading) and negative (e.g., increased inflammation, fewer mesenchymal stem cells directed to osteoblasts) effects [2,13]. Therefore, it is possible that increased fat mass in NWO could still have a net negative effect on overall bone health. Lastly, we observed higher lean mass percent in Con versus NWO, which suggests our NWO group was less physically active (in particular less resistance exercise) than Con. It seems plausible that lower overall physical activity may be related to both increased body fat percent and lower whole-body BMD in NWO, so future work should attempt to disentangle the unique impact of NWO status versus lack of physical activity on body composition and bone health in adults with NWO.

Although we are the first to report altered indicators of bone homeostasis in NWO, this study is not without its limitations. First, given our relatively small sample, our findings should be confirmed in larger samples, ideally separated by sex. Moreover, given the nature of this secondary analysis, we were unable to perform DXA scans of at-risk sites (i.e., femoral neck, lumbar spine). Additionally, we did not measure all established serum indicators of bone health and future work should examine markers such as carboxy-terminal collagen crosslinks (CTX), tartrate-resistant acid phosphatase (TRAP), procollagen type I N-terminal propeptide (PINP) in NWO. Lastly, our sample primarily consisted of non-Hispanic white participants, so this study should be replicated in other racial and ethnic groups.

## 5 Conclusion

In summary, we present initial evidence that NWO is related to low whole-body BMD alongside some serum markers associated with bone resorption. Despite the majority of research focusing on higher body fat and lower lean mass in this population, poor bone health may be another adverse trait in NWO and warrants further study.

## Author contributions

BHK: Conceptualization, Methodology, Formal analysis, Investigation, Data curation, Writing – original draft, Writing – review & editing, Visualization, Project administration, Funding acquisition. CMS: Methodology, Investigation, Writing – review & editing. ARM: Methodology, Investigation, Writing – review & editing. SMH: Methodology, Investigation, Writing – review & editing. EHC: Methodology, Investigation, Writing – review & editing. SRE: Conceptualization, Methodology, Investigation, Writing – review &editing, Supervision, Project administration, Funding acquisition.

## Ethical review

This work was carried out in accordance with the Declaration of Helsinki and approved by the Oklahoma State University Institutional Review Board. All participant provided written informed consent before completing the study.

## Source of funding

American Society for Nutrition Mars Inc. Predoctoral Fellowship, Barbara K. Pass Nutritional Sciences Research Grant.

## Declaration of competing interest

The authors declare that they have no known competing financial interests or personal relationships that could have appeared to influence the work reported in this paper.
